# Boosting Transport Kinetics of Ions and Electrons Simultaneously by Ti_3_C_2_T_*x*_ (MXene) Addition for Enhanced Electrochromic Performance

**DOI:** 10.1007/s40820-020-00544-9

**Published:** 2020-11-06

**Authors:** Wenting Wu, Huajing Fang, Hailong Ma, Liangliang Wu, Wenqing Zhang, Hong Wang

**Affiliations:** 1 State Key Laboratory for Mechanical Behavior of Materials, School of Material Science and Engineering, Xi’an Jiaotong University, Xi’an, 710049 People’s Republic of China; 2 School of Electronic and Information Engineering and State Key Laboratory for Mechanical Behavior of Materials, Xi’an Jiaotong University, Xi’an, 710049 People’s Republic of China; 3 Department of Physics, Southern University of Science and Technology, Shenzhen, 518055 People’s Republic of China; 4 Department of Materials Science and Engineering, Southern University of Science and Technology, Shenzhen, 518055 People’s Republic of China; 5 Shenzhen Engineering Research Center for Novel Electronic Information Materials and Devices, Southern University of Science and Technology, Shenzhen, 518055 People’s Republic of China

**Keywords:** Electrochromic, Mxene, Transport kinetics, Ionic conductivity, Tungsten oxide

## Abstract

Electrochromic technology plays a significant role in energy conservation, while its performance is greatly limited by the transport behavior of ions and electrons. Hence, an electrochromic system with overall excellent performances still need to be explored. Initially motivated by the high ionic and electronic conductivity of transition metal carbide or nitride (MXene), we design a feasible procedure to synthesize the MXene/WO_3−*x*_ composite electrochromic film. The consequently boosted electrochromic performances prove that the addition of MXene is an effective strategy for simultaneously enhancing electrons and ions transport behavior in electrochromic layer. The MXene/WO_3−*x*_ electrochromic device exhibits enhanced transmittance modulation and coloration efficiency (60.4%, 69.1 cm^2^ C^−1^), higher diffusion coefficient of Li^+^ and excellent cycling stability (200 cycles) over the pure WO_3−*x*_ device. Meanwhile, numerical stimulation theoretically explores the mechanism and kinetics of the lithium ion diffusion, and proves the spatial and time distributions of higher Li^+^ concentration in MXene/WO_3−*x*_ composite electrochromic layer. Both experiments and theoretical data reveal that the addition of MXene is effective to promote the transport kinetics of ions and electrons simultaneously and thus realizing a high-performance electrochromic device. This work opens new avenues for electrochromic materials design and deepens the study of kinetics mechanism of ion diffusion in electrochromic devices.

**Electronic supplementary material:**

The online version of this article (10.1007/s40820-020-00544-9) contains supplementary material, which is available to authorized users.

## Highlights

- MXene/WO_3−*x*_ composite film with excellent electrochromic performances was fabricated for the first time.
- The addition of MXene is an effective strategy for simultaneously enhancing electron and ion transport behaviors in the electrochromic layer.
- The kinetics of ion diffusion in electrochromic devices are studied in depth based on both experiment and simulation.

**Electronic supplementary material:**

The online version of this article (10.1007/s40820-020-00544-9) contains supplementary material, which is available to authorized users.

## Graphical abstract

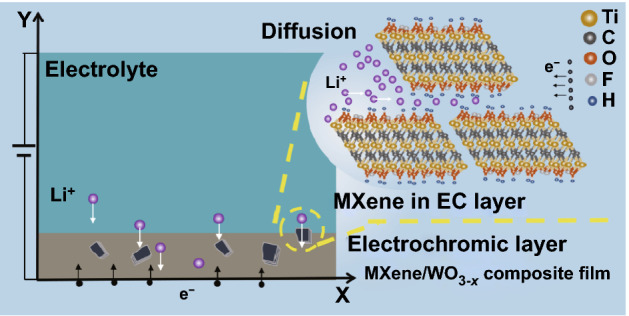

## Introduction

With the rapid development of industrial society, the energy crisis and environmental deterioration have become serious problems that humans have to face [1]. As the building energy consumption accounts for almost 40% of the total energy consumption of the whole society, it has become the primary goal of energy conservation [2, 3]. Electrochromic technology, which can be applied as smart windows, plays an important role in this regard because of the ability for dynamically controlling the indoor temperature and natural lighting of the building [4, 5]. The core components of electrochromic technology are electrochromic materials whose color and transmittance can be reversibly changed upon an electric bias. In recent years, a variety of electrochromic materials have been widely studied [6–11]. Among them, tungsten oxide (WO_3−*x*_) is considered to be one of the most suitable candidates for commercialization, due to advantages of significant color change, non-toxicity, easy preparation, and resistance to ultraviolet radiation [12–14]. The electrochromic mechanism of tungsten oxide is widely accepted as follow [15]:

1 $${\text{WO}}_{{{3} - x}} \left( {\text{transparent state}} \right) \, + \, \alpha {\text{M}}^{{{\text{k}} + }} + \, \alpha {\text{ke}}^{ - } \leftrightarrow {\text{ M}}_{\alpha } {\text{WO}}_{{{3} - x}} \left( {\text{colored state}} \right)$$

M^k+^ stands for monovalent ions such as H^+^, Li^+^, and Na^+^, or multivalent ions (e.g., Mg^2+^, Zn^2+^, Al^3+^) [16–18]. It is clear that electrochromism of WO_3−*x*_ is essentially an interface electrochemical reaction, demanding double injection and extraction of cations and electrons. Generally, excellent electrochromic performances include the following four parameters: large transmittance modulation, good cycling performance, short response time and high coloration efficiency. However, large transmittance modulation means a large number of ions inside the electrochromic materials which is to some extent opposite to the short response time. It can also be reasonably explained when considering doping in electrochromic materials: shallow doping provides a large number of carriers to accommodate ions (large transmittance modulation) but reduces the recombination rate (long response time); deep doping shows the opposite [19]. In addition, the short response time is more related to loose structured electrochromic materials. The structure in turn greatly reduces the stability of cycling performance. Therefore, an electrochromic system with excellent overall performances is urgently needed but difficult to achieve.

Starting with the fundamental electrochemical reaction, electrochromic performances of the WO_3−*x*_ depend on the transport rate of the cations and electrons as well as the ability to accommodate the cations in the film. Different advanced strategies have been studied to improve electrochromic performances such as the combination of crystalline and amorphous WO_3−*x*_ [20, 21], regulation of nanotopography [6, 22, 23], element doping [18, 24, 25], and construction of composite materials [26, 27]. On the one hand, the structure construction of tungsten oxide almost concentrates on the transport behavior of cations. For example, Lee’s group has fabricated the nanostructured porous film with large transmittance modulation, which improves the ionic conductivity by facilitating the electrolyte penetration and shortening the ionic diffusion [23]. Huo et al. [28] prepared the hexagonal/amorphous tungsten oxide core/shell nanorod arrays with enhanced electrochromic and pseudocapacitive properties, where the structure provides effective channels and more active sites to improve the ionic transport. Also, the element doping introduces the oxygen vacancies which is beneficial to ionic conductivity. On the other hand, hybrid tungsten oxide film with other nanomaterials, like reduced graphene oxide (rGO) [29–31], carbon nanotubes (CNT) [32, 33], and g-C_3_N_4_ [34] have been investigated with enhanced electrochromic performance. These additives all own high-electronic conductivity and large surface area that the transport of electrons is facilitated. Restricted by the intrinsic characteristics of these materials, such as the interlayer space and energy barrier [35, 36], the improvement in ion intercalation and diffusion inside the electrochromic film is limited, even they can hinder the intercalation pathway causing the loss of ionic insertion capability in some cases [32, 37]. There is nearly no simple and effective strategy to simultaneously boost the transport kinetics of electrons and ions in tungsten oxide thin films up to now.

The emergence of a new two-dimensional material, transition metal carbide or nitride (MXene) represented by Ti_3_C_2_T_*x*_ (T=F, OH, etc.,), provides an opportunity to solve the longstanding problem in electrochromic technology. Since being first reported in 2011, MXene has made many important breakthroughs in the fields of batteries, supercapacitors, and catalysis [38–42]. This material has a metal-like electronic conductivity which can be used as transparent conductor in electronic and sensor applications [43]. And the layer structure is maintained by weak van der Waals force and the layer spacing is large enough to provide highly ordered two-dimensional nanochannels for ion transport [44, 45]. In short, MXene is such materials with both high ionic and electronic conductivity. This work is initially motivated by the excellent properties of MXene, thereby, we design the MXene/WO_3−*x*_ composite film as electrochromic material for the first time. A feasible procedure is adopted to synthesize this composite film, and the boosting electrochemical kinetics is proven with enhanced electrochromic performances. Compared with pure WO_3−*x*_ film, the MXene/WO_3−*x*_ composite film displays a larger transmittance modulation, a remarkable coloration efficiency, and a much better cycling stability. Meanwhile, the electrochemical reaction kinetics behaviors have been unraveled by numerical stimulation, which further prove that the increased transport rate of the cations is due to the MXene addition.

## Experimental

### Preparation of Electrochromic Films and Devices

The tungsten oxide precursor was fabricated according to a previous work [46]. Briefly, tungsten power (99.8%, Sinopharm, China) was added to hydrogen peroxide (30% H_2_O_2_, Sinopharm, China) and reacted in an ice-water bath. After the supernatant was evaporated and dried, the residue was then dispersed ultrasonically and filtered, resulting in the clear yellow tungsten oxide precursor exhibiting the Tyndall effect, as shown in Fig. S1a. The MXene aqueous solution was fabricated via etching, intercalation and delamination (detailed information is shown in Supporting Information S1). Then the hybrid precursor of MXene (Ti_3_C_2_T_*x*_) and tungsten oxide was obtained by adding MXene aqueous solution (20 μL) into the 2 mL tungsten oxide precursor with a volume ratio of 1:100, then we produced a brownish green solution (Fig. S1b).

The 2.5 × 2.5 cm^2^ FTO glasses were treated by ultrasonic cleaning with acetone, ethanol and deionized water for 15 min each. Then the pure WO_3−*x*_ and MXene/WO_3−*x*_ films were formed by spin coating at 1000 rpm for 20 s with corresponding precursor for repeated six times. These films were annealed at 200 °C for 40 min. The LiClO_4_ (Sinopharm, China) salt was dried and then dissolved in the propylene carbonate (PC) and dimethyl carbonate (DMC) (1:1 in volume) (Aladdin, China). To fabricate the device, the FTO glass with deposited electrochromic film, the LiClO_4_: PC and DMC electrolyte and another FTO glass electrode were assembled, creating a sandwich structure. Between the spaces of two FTO substrates, the device was sealed by the annular transparent silicone in case of leakage.

### Characterization

Raman spectra of the MXene, pure WO_3−*x*_ and MXene/WO_3−*x*_ composite films were performed by a Raman spectroscopy (LabRAM HR Evolution) at the excitation wavelength of 532 nm. The X-ray photoelectron spectroscopy (XPS, ESCALAB Xi^+^) was used to confirm the groups of MXene. The morphology of the pure WO_3−*x*_ and MXene/WO_3−*x*_ films were recorded by SEM (FEI Quanta 250 FEG). The TEM image of MXene/WO_3−*x*_ composite film was investigated by field emission transmission electron microscope (TEM, JEOL JEM-F200 (HR). Conductivity of the LiClO_4_ in PC and DMC electrolyte was measured by a conductivity meter (DDS-307A, REX) which was calibrated with conductivity standard KCl solutions at 25 °C. Measurements of the transmittance of the electrolyte, films and devices were performed using a spectrophotometer (Mapada V-1600PC). Chronoamperometry curve was obtained by a digital sourcemeter (Keithley 2410). Cyclic voltammetry (CV) measurements were carried out on a Zahner electrochemical workstation (Zennium pro).

## Results and Discussion

### Structure Characterization of MXene/WO_3−*x*_ Composite Film

**Fig. 1:**
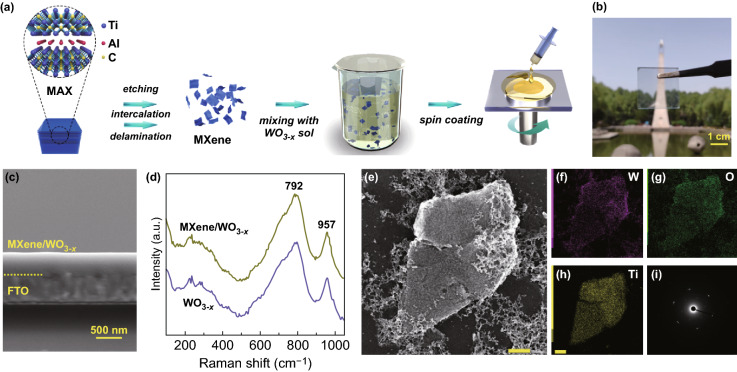
**a** Schematic diagram depicting the fabrication process of MXene/WO_3−*x*_ films. **b** A photo of the prepared MXene/WO_3−*x*_ film on FTO glass (2.5 × 2.5 cm^2^). The scale bar is 1 cm. **c** Cross-sectional SEM of the MXene/WO_3−*x*_ film. **d** Raman spectra of the pure WO_3−*x*_ and MXene/WO_3−*x*_ films. **e** TEM image, **f–h** EDS mapping and **i** SAED of the MXene/WO_3−*x*_ films. Scale bar in figure **e** and **h** represents the length of 200 nm

As shown in Fig. 1a, the schematic diagram presents the fabrication of MXene (Ti_3_C_2_T_*x*_)/tungsten oxide films. Then we measured the XPS, TEM, and Raman spectra of MXene to identify the chemical structure and morphology of the MXene. The TEM images of the MXene from top view present the separated two-dimensional nanoflakes (Fig. S2). The XPS results of the MXene show the existence of H, F, O, C, Ti elements and presence of -OH and -F functional groups (Fig. S3). Raman results show the corresponding surface chemistry and structure of MXene (Fig. S4).

As illustrated in experimental section, the transparent composite film is deposited on FTO substrate (Fig. 1b). Considering that a small amount of MXene solution is added into the WO_3−*x*_ precursor, the thickness and crystal structure of MXene/WO_3−*x*_ film do not alter compared with those of pure WO_3−*x*_ film. As shown in Fig. 1c, the MXene/WO_3−*x*_ composite film is seen to be deposited seamlessly on FTO substrate and the thickness is about 430 nm (Fig. 1c), so is the thickness of pure WO_3−*x*_ film (Fig. S5). And the Raman spectra characterization also presents similar structure features between pure WO_3−*x*_ film and MXene/WO_3−*x*_ film as expected. Beside the immanent character of FTO substrates at 235 cm^−1^, the strong peaks at 792 and 957 cm^−1^ identified in both pure WO_3−*x*_ and MXene/WO_3−*x*_ films are the typical features of amorphous WO_3−*x*_. It is assigned to the stretching mode of W–O bonds and terminal W = O double bonds, respectively [47].

To further confirm the morphology and chemical composition of the MXene/WO_3−*x*_ composite film, we have firstly carried out the SEM measurement. Surface morphology (Fig. S6) shows that the small MXene nanoflakes are distributed in the amorphous WO_3−*x*_ substrate, where the size varies from tens to hundreds of nanometers. Furthermore, the morphology and element distribution of MXene/WO_3−*x*_ film have been measured by TEM and EDS mapping (Fig. 1e-h). In the detected area, the MXene are evenly distributed over the WO_3−*x*_. The multiple diffractions image is observed in the selected area electron diffraction (SAED) pattern in Fig. 1i. It consists of the diffraction halo from amorphous WO_3−*x*_ and sharp diffraction spots arising from MXene. Therefore, it can be concluded that MXene nanoflakes are closely and uniformly embedded in amorphous WO_3−*x*_ [48].

### Configuration and Performances of the Electrochromic Devices

**Fig. 2:**
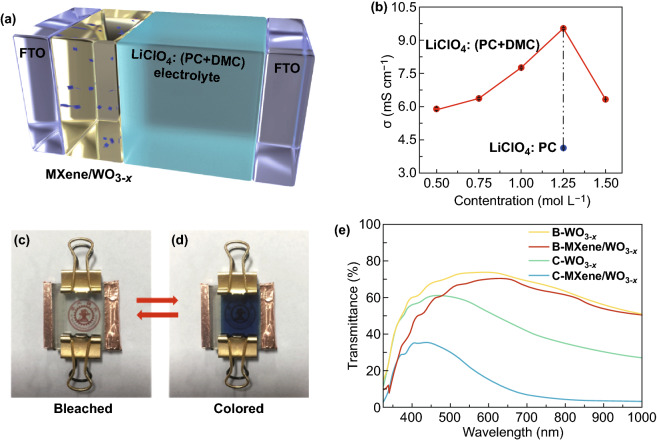
**a** Schematic illustration of the electrochromic device based on the MXene/ WO_3−*x*_ film. **b** Conductivity of the electrolytes at room temperature with different LiClO_4_ concentration. Digital photos of the electrochromic device based on the MXene/WO_3−*x*_ film in **c** colored state and **d** bleached state. **e** Transmittance spectra of pure WO_3−*x*_ and MXene/WO_3−*x*_ electrochromic devices in colored and bleached states

In Fig. 2a, the configuration of electrochromic device is schematically shown. To obtain excellent performances, improvements in both electrochromic material and electrolyte layer should be addressed [49]. The electrochromic material is optimized through MXene addition at the optimal ratio of 20 μL (MXene solution): 2 mL (tungsten oxide precursor). Also, the thickness (fabrication layer number of 6) of electrochromic film is also optimally regulated. The effects of MXene addition ratio and thickness on electrochromic performances are investigated and discussed in Figs. S7–S10.

At the same time, the electrolyte optimization is settled with the outstanding conductivity and appropriate viscosity, which facilitates ion transport during the electrochemical process. Herein, we use LiClO_4_ with mixture solvents of PC and DMC as the electrolyte, as the lithium ions play a vital role in electrochromic applications [50, 51]. The binary solvent system of PC and DMC exhibits better characteristics in electrochemical performance [52, 53], where the addition of DMC solvent in binary solvent system not only reduces high viscosity of PC solvent [53], but also achieves higher ionic conductivity and transmittance (Fig. S11). It’s shown to be more suitable for the realization of transparent electrochromic devices. Furthermore, the conductivity maxima (9.5 mS cm^−1^) lies in 1.25 mol L^−1^ of LiClO_4_ concentration by investigating the optimal concentration (Fig. 2b), which takes a great advantage than Li^+^-based electrolyte in previous studies [54]. Within the range of the optimal concentration, conductivity is raised by the increasing salt concentration, along with a larger number of charge carriers. Whereas in the concentration higher than the optimum, conductivity is decreased by the reduced number of valid charge carriers and restricted mobility of the charge carriers [55]. It can be explained more precisely: (1) higher salt concentration increases the ion-pairing between molecules, causing the reduced number of valid charge carriers; (2) higher salt concentration results in formation of higher aggregated ions, which increases the migration resistance and restricts the mobility of charge carriers.

Next, the electrochromic device of MXene/WO_3−*x*_ electrochromic film with the optimal electrolyte is fabricated. By applying positive and negative bias of 3 V, the double injections and extractions of electrons and Li^+^ in the electrochromic film are reversible progressed. The specific equation is as following [56]:

2 $${\text{WO}}_{3 - x} + ye^{ - } + \, y{\text{Li}}^{ + } \leftrightarrow {\text{ Li}}_{y} {\text{WO}}_{3 - x} \left( {0 \, < y < \, 1} \right)$$

Also, MXene can be functioned as a host for reversible Li^+^ injections and extractions [57], which is also involved in the electrochromic reaction. On account of a small number of MXene nanoflakes in the composite film, we only exhibit the electrochromic mechanism of tungsten oxide in Eq. (2).

The MXene/WO_3−*x*_ electrochromic device shows visually identifiable colored and bleached states. The bleached state (Fig. 2c) clearly exhibits the logo behind and a blue and uniform colored state is presented in Fig. 2d. Furthermore, the transmittance spectra of MXene/WO_3−*x*_ electrochromic device is compared with that of pure WO_3−*x*_ device in Fig. 2e. The bleaching state of the two devices owns transmittance of 71% and 69.6% at 660 nm. The transimttance of bleached device is influenced by the transmittance of FTO substrates and electrolyte layer, especially in the infrared band (lower than 60%) (Fig. S12). The decreasing transmittance of less than 2% is owning to balck MXene addition. Notablely, the transmittance modulation of MXene/WO_3−*x*_ electrochromic device (*λ* = 660 nm, 60.4%) is far larger than that of pure WO_3−*x*_ electrochromic device (*λ* = 660 nm, 25.3%), which implies that more lithium ions are intercalated into the MXene/WO_3−*x*_ electrochromic film. To further dig out the contribution of MXene, we explore the performance of the same amount of MAX solution added in WO_3−*x*_. The MAX/WO_3−*x*_ device exhibits almost the same transmittance modulation as the pure WO_3−*x*_ device (Fig. S13), ensuring the effect of MXene addition on the electrochromic performance is due to the layered structure rather than the chemical composition. The more intercalated lithium ions can be contributed to the provided nanochannels and active sites in the two-dimensional layered structure of MXene. At the same time, MXene, as the electrochromic material [58], produce synergistic benefits with tungsten oxide in enhancing electrochromic properties.

**Fig. 3:**
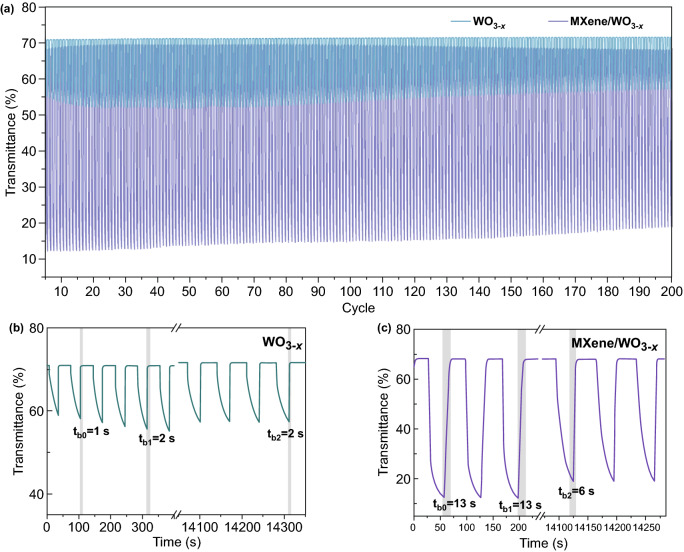
**a** Cyclic stability of pure WO_3−*x*_ and MXene/WO_3−*x*_ electrochromic devices. **b** Amplified in situ transmittance curve of the pure WO_3−*x*_ device. **c** Amplified in-situ transmittance curve of the MXene/WO_3−*x*_ device

To study the stability of pure WO_3−*x*_ and MXene/WO_3−*x*_ electrochromic devices, the cycling performance is evaluated by repeated switching test with coloring at 3 V for 30 s and bleaching at 3 V for 40 s (Fig. 3a). Compared with that of pure WO_3−*x*_ device, significant larger transmittance modulation is found during the whole 200 cycles in MXene/WO_3−*x*_ device. It’s worth noting that they all shows stable transmittance in bleached state, which can be explained that only shallow traps occur during the lithium ion intercalation process, only degrading the colored state [59]. And traps can cause the structural damage of electrochromic materials, leading to the degradation of transmittance modulation and weakening the stability. As seen from the amplified curves of the two devices, the transmittance modulation slightly decreases from 15.11 to 14.15%, and maintains the stable bleaching time 2 s (Fig. 3b) in pure WO_3−*x*_ device. For the MXene/WO_3−*x*_ device, the initial transmittance modulation of 56% keeps stable for a long time, and remains 48.8% (87.2% of its initial value) after 200 cycles (as long as 14,000 s) in Fig. 3c. The bleaching time is shortened from 13 to 6 s, because the trapped ions reduce the number of lithium ions involving in the extraction process. Overall, the MXene/WO_3−*x*_ electrochromic device shows excellent stability performance over the 200 cycles.

**Fig. 4:**
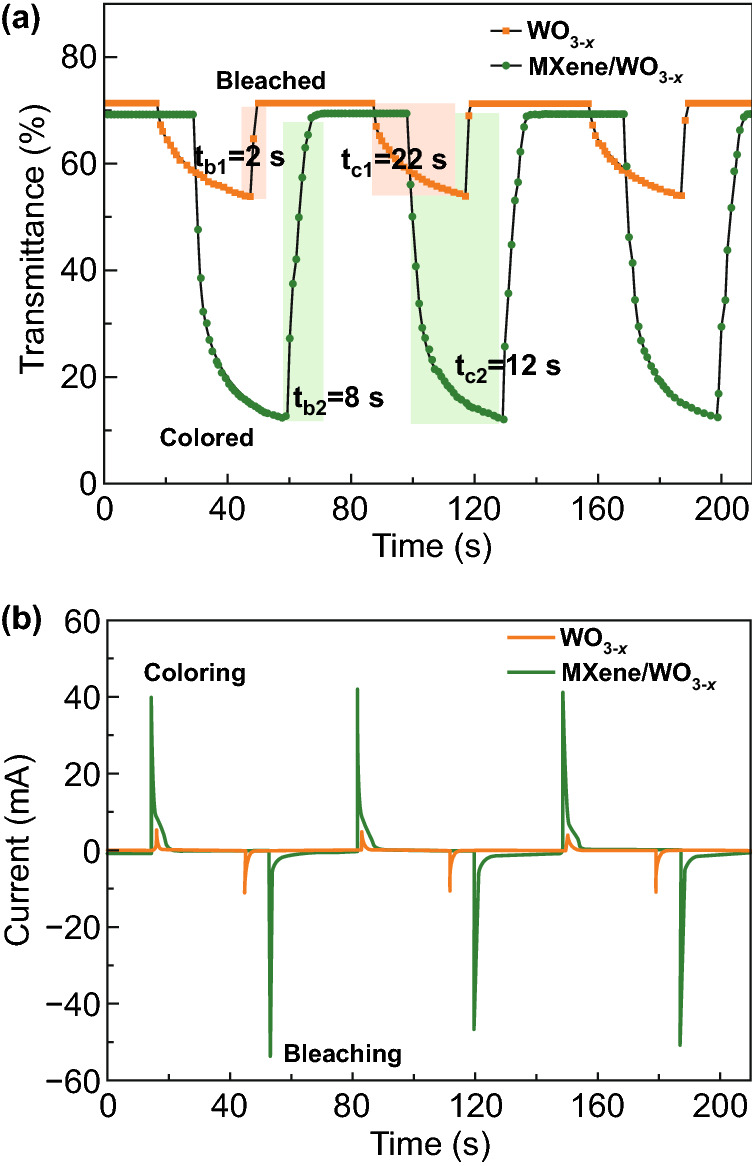
**a** In-situ transmittance curves of the electrochromic devices based on pure WO_3−*x*_ and MXene/WO_3−*x*_ films at 660 nm. **b** Corresponding chronoamperometry curves of the two electrochromic devices

Response time is one of the key properties of electrochromic device, which is defined as the time for 90% of transmittance modulation during coloring/bleaching. Figure 4a shows the stable optical transmittance response of the pure WO_3−*x*_ and MXene/WO_3−*x*_ electrochromic devices at 660 nm with coloring for 30 s and bleaching for 40 s of per step. From the platform at the bleached state, both devices are fully bleached with sufficient time to ensure that as many ions as possible are extracted from the electrochromic materials. Pure WO_3−*x*_ device exhibits response time of coloring (22 s) and bleaching (2 s) of small transmittance modulation (17.5%). And in MXene/WO_3−*x*_ device, the coloring and bleaching response time are found to be 12/8 s with the resultant large transmittance modulation (56.82%). Based on the transport mechanism of MXene, it provides the surface channels and interlayer channels [60, 61], increasing the total lithium ion concentration in the electrochromic layer during the same coloring time. Thus, increased lithium ions cause longer bleaching time of transport in composite film when compared with that of pure WO_3−*x*_ film.

As another important property, the coloration efficiency (CE) refers to the changes in optical density (ΔOD) per unit charge density (ΔQ) inserted into or extracted from the electrochromic film. It can be calculated from the formula below [29]:

3 $${\text{CE }} = \, \Delta {\text{OD}}/\Delta {\text{Q }} = {\log}\left( {T_{{\text{b}}} /T_{{\text{c}}} } \right) \, / \, \Delta {\text{Q}}$$

where *T*_b_ and *T*_c_ represent the bleached and colored transmittances, respectively, ΔQ can be obtained from current integration of the corresponding chronoamperometry curves (Fig. 4b).

The CE of MXene/WO_3−*x*_ device is 69.1 cm^2^ C^−1^, which is higher than the value of pure WO_3−*x*_ device (42.3 cm^2^ C^−1^). Also, the peak currents of MXene/WO_3−*x*_ are far higher than that of pure WO_3−*x*_ in the curves. The higher CE and current value indicate more electrochromic materials are activated and more lithium ions and electrons are extracted and inserted [62], which is confirmed by the MXene addition.

**Fig. 5:**
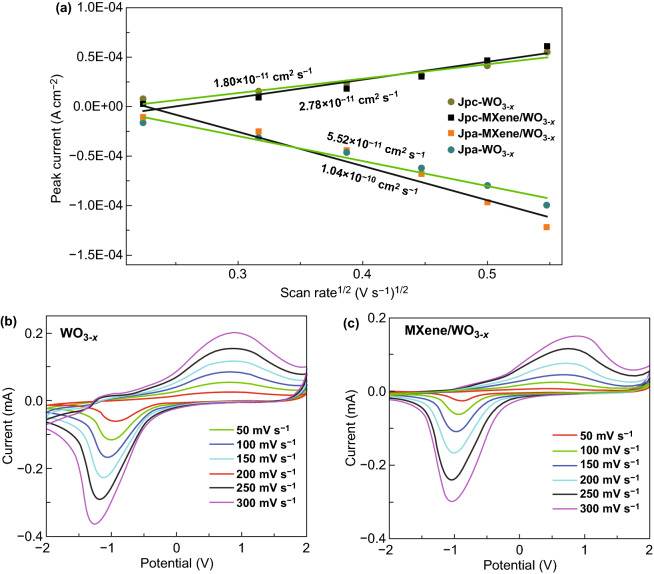
**a** Diffusion coefficient of WO_3−*x*_ film with and without MXene addition. Cyclic voltammogram of the device based on **b** pure WO_3−*x*_ film and **c** MXene/WO_3−*x*_ film

As far as we know, the intercalation chemistry takes a dominant impact in electrochromic devices. Then the electrochemical analysis from the cyclic voltammogram (CV) further verifies fast lithium ion diffusion in MXene/WO_3−*x*_ device. As illustrated in Fig. 5a, the cathodic and anodic peak current densities (*J*_pc_ and *J*_pa_) of the WO_3−*x*_ and MXene/WO_3−*x*_ electrochromic devices as a function of (scan rate)^1/2^ identify the lithium ion insertion/extraction coefficient. These current values have been obtained at various scan rate of 50, 100, 150, 200, 250, 300 mV s^−1^ between −2 and 2 V in Fig. 5b, c, then the diffusion coefficients of Li^+^ are calculated by the Randles-Servcik equation:

4 $$J_{{\text{p}}} = {2}.{72} \times {1}0^{{5}} n^{{{3}/{2}}} {\text{D}}^{{{1}/{2}}} {\text{C}}_{0} v^{{{1}/{2}}}$$

where D is the diffusion coefficient in the unit of cm^2^ s^−1^, *J*_p_ is peak current density (A cm^−2^), n is the number of electrons assumed to be 1 for Li^+^, *C*_0_ is the concentration of electrolyte solution (mol cm^−3^) and *v* is the scan rate (V s^−1^).

The calculated diffusion coefficients of Li^+^ insertion are 2.78 × 10^−11^ and 1.80 × 10^−11^ cm^2^ s^−1^ with and without MXene addition, respectively. Also, diffusion coefficients of Li^+^ extraction are 1.04 × 10^−10^ and 5.52 × 10^−11^ cm^2^ s^−1^ with and without MXene addition, which show the quantitative agreement with the previous studies [63, 64]. The lithium ion insertion/extraction speed into the electrochromic film is evidently enhanced by the addition of MXene.

Overall, the MXene/WO_3−*x*_ film with above electrochromic performances exhibit a competitive advantage when compared with those of hybrid tungsten oxide film with other nanomaterials additions, as shown in Table 1. MXene simultaneously enhances the transport of electrons and ions in electrochromic reaction. It can be ascribed to three explanations: (1) MXene with excellent electronic conductivity promotes the electrons transport ability in the electrochromic layer [65]. (2) The lateral layer spaces in the structure of MXene are large enough, which can be functioned as nanochannels to facilitate ion transport [66]. (3) The interface of the hybrid electrochromic material MXene/WO_3−*x*_ allows more active sites and lower activation energy in electrochromic reaction [67, 68].

**Table 1:** Electrochromic performances of tungsten oxide nanocomposite film

Materials	Fabrication method	Transmittance modulation	Switching speed *t*_c_/*t*_b_ (s)	Coloration efficiency (cm^2^/C)	Electrolyte	Cycling performance (retaining)	Refs
MXene/WO_3−*x*_	Spin coating	60.4% @660 nm	12/8	69.1	1.25 M LiClO_4_-PC + DMC	87.2%/200cycles	This work
MWCNTs/WO_3−*x*_	Spin coating	69% @550 nm	8/1	29.9	1 M LiClO_4_-PC	80.6%/200cycles	[32]
CS-CNTs/WO_3_	Layer-by-layer Dip coating	13.5%@800 nm	1.9/1	79.2	1 M LiClO_4_-PC	37.1%/200cycles	[33]
RGO/WO_3_	Spin coating	59.6% @633 nm	9.5/7.6	75.3	1 M LiClO_4_-PC	150cycles	[30]
RGO/WONWS	Solvothermal	53% @600 nm	1.5/1.2	116.7	0.5MLiClO_4_-PC	—	[31]
GO/WO_3_·H_2_O	Sonochemical	10% @632 nm	2/3	67.66	1 M LiClO_4_-PC	—	[34]
g-C_3_N_4_/WO_3_·H_2_O	Sonochemical	10% @632 nm	5/5.5	60.13	1 M LiClO_4_-PC	—	[34]
MoS_2_/WO_3_	Spin coating	75% @700 nm	11/8	51.2	0.5 M LiClO_4_-0.05 M Fc-PC	—	[72]

“—” means data not available

*MWCNT* multiwalled carbon nanotube, *CS* chitosan, *WONWS* tungsten oxide nanowires

### Simulation of Ion diffusion in Electrochromic Layer

**Fig. 6:**
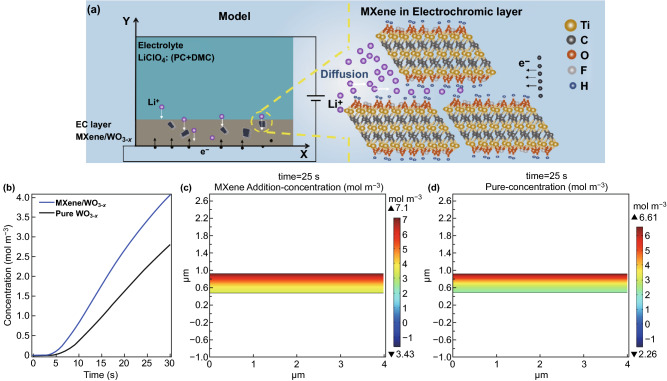
**a** Model of the simulation and specific kinetic process of the lithium ion diffusion. **b** Time distribution of Li^+^ in pure WO_3−*x*_ film and MXene/WO_3−*x*_ film. Spatial distribution (time = 25 s) of Li^+^ in **c** MXene/WO_3−*x*_ film and **d** pure WO_3−*x*_ film

In order to verify the contribution of MXene in electrochromic reaction, we further explore the kinetics mechanism of lithium ion diffusion inside the electrochromic film by using finite element analysis (COMSOL Multiphysics). As shown in Fig. 6a, a simplified two-dimensional (2D) model is created to simulate the coloration process in electrochromic device. The specific kinetic process of the lithium ion diffusion is presented here. A widely accepted theory emphasizes that the ion diffusion in electrochromic film is a rate-controlling step for the kinetics [69]. The layer spacing of MXene in electrochromic film provides nanochannels for Li^+^ diffusion, it then be beneficial for the combination with electrons and tungsten oxide to produce redox reaction [70, 71]. As a result, it can effectively enhance the intercalation rate with color change phenomenon. In this simulation, the Secondary Current Distribution module and the Transport of Diluted Species module are coupled to study the lithium ion transport behaviors between the MXene/WO_3−*x*_ and pure WO_3−*x*_ electrochromic devices. After applying the corresponding potential in the boundary, the electrochemical reaction is then produced at the interface between the layers. The simulation results of Fig. 6b present Li^+^ concentration variations during the whole coloration time (30 s) at the same Y-axis of the MXene/WO_3−*x*_ and pure WO_3−*x*_ electrochromic film, where MXene/WO_3−*x*_ composite electrochromic film shows higher Li^+^ concentration than that of the pure WO_3−*x*_ film. The increasing concentration also indicates the dynamic ion diffusion process inside the electrochromic layer over time. Moreover, the 2D spatial distribution results of Fig. 6c, d shows that the MXene/WO_3−*x*_ film accommodates more lithium ions than the pure WO_3−*x*_ film in whole region at any coloration moment (Li^+^ dynamic diffusion in whole coloration time is shown in Fig. S14). The results shown above provide a clear mechanism study for the contribution of MXene in time and spatial distribution, which agree well with the experimental results. This study reveals a deeper understanding of lithium intercalation kinetics and the diffusion process in the electrochromic film.

## Conclusions

In summary, we creatively fabricate the MXene/WO_3−*x*_ composite film applied in electrochromic devices for the first time. The addition of MXene in tungsten oxide thin films is a simple and effective strategy to simultaneously boost the transport kinetics of electrons and ions. Compared with that of pure WO_3−*x*_ device, the transmittance modulation and coloration efficiency have been improved after combination. Such enhanced electrochromic properties are attributed to the metal-like electronic conductivity and two-dimensional layered structure of MXene and the resultant improved electrochemical activity. Moreover, fast ion transport kinetics and cycling stability over 200 cycles are identified and analyzed in Mxene/WO_3−*x*_ device, which present higher diffusion coefficients of Li^+^ insertion and extraction. Numerical stimulation further proves the spatial and time distributions of higher Li^+^ concentration in the Mxene/WO_3−*x*_ electrochromic layer. Both experiments and theoretical aspects demonstrate the contribution of MXene in high-performance electrochromic device by boosting the transport kinetics of ions and electrons simultaneously. It shows a paradigm on rational design of electrochromic materials and provides new avenues to explore the kinetics mechanism of lithium ion diffusion inside the electrochromic film.

## Electronic supplementary material

Below is the link to the electronic supplementary material.

Supplementary file1 (PDF 889 kb)
