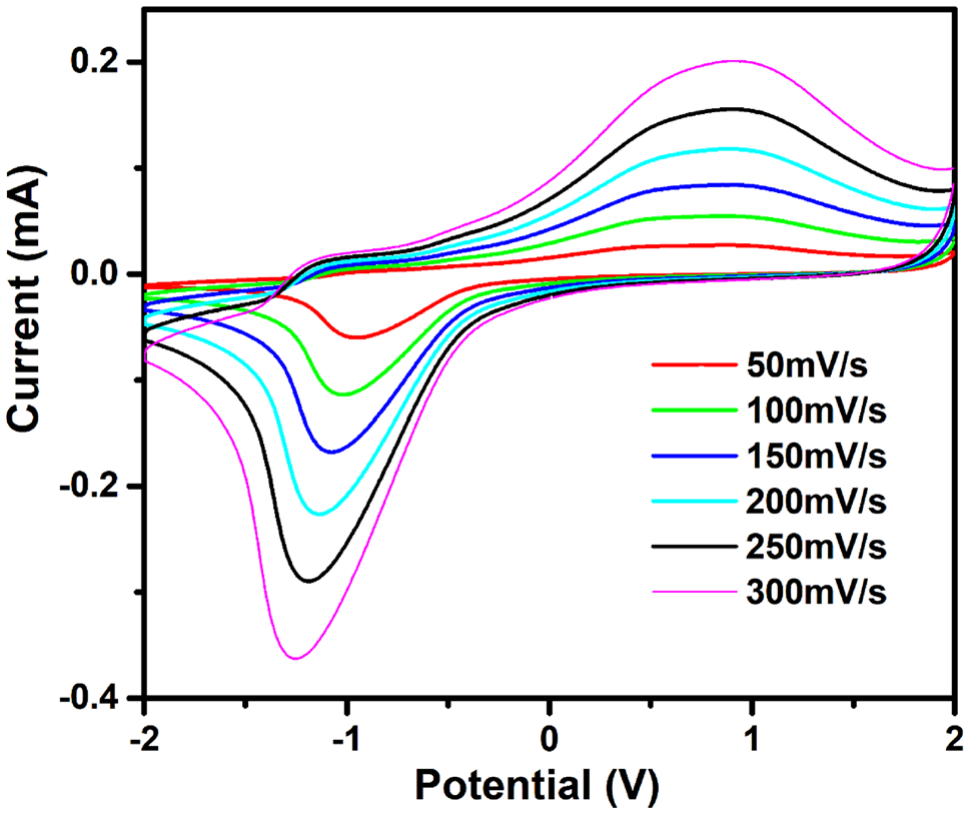# Correction to: Boosting Transport Kinetics of Ions and Electrons Simultaneously by Ti_3_C_2_T_*x*_ (MXene) Addition for Enhanced Electrochromic Performance

**DOI:** 10.1007/s40820-021-00739-8

**Published:** 2021-10-21

**Authors:** Wenting Wu, Huajing Fang, Hailong Ma, Liangliang Wu, Wenqing Zhang, Hong Wang

**Affiliations:** 1 State Key Laboratory for Mechanical Behavior of Materials, School of Material Science and Engineering, Xi’an Jiaotong University, Xi’an, 710049 People’s Republic of China; 2 School of Electronic and Information Engineering and State Key Laboratory for Mechanical Behavior of Materials, Xi’an Jiaotong University, Xi’an, 710049 People’s Republic of China; 3 Department of Physics, Southern University of Science and Technology, Shenzhen, 518055 People’s Republic of China; 4 Department of Materials Science and Engineering, Southern University of Science and Technology, Shenzhen, 518055 People’s Republic of China; 5 Shenzhen Engineering Research Center for Novel Electronic Information Materials and Devices, Southern University of Science and Technology, Shenzhen, 518055 People’s Republic of China

## Correction to: Nano-Micro Lett (2021) 13:20 10.1007/s40820-020-00544-9

The original version of this article unfortunately contained some mistakes in figure. The authors found that explanation of the data lines in Fig. 5b is wrong.

The corrected version of Fig. 5b is given below: