# Supplementary material for: Boosting Transport Kinetics of Ions and Electrons Simultaneously by Ti3C2Tx (MXene) Addition for Enhanced Electrochromic Performance
Source: Nanomicro Lett. 2020 Nov 6;13:20. doi: 10.1007/s40820-020-00544-9 (PMC8187520; doi:10.1007/s40820-020-00544-9)
Supplement: Supplementary file 1 — Supplementary file1 (PDF 889 kb) [file 40820_2020_544_MOESM1_ESM.pdf]

Supporting Information for

Boosting Transport Kinetics of Ions and Electrons Simultaneously by $Ti_3C_2T_x$ (MXene) Addition for Enhanced Electrochromic Performance

Wenting Wu[1], Huajing Fang[1, *], Hailong Ma[1], Liangliang Wu[2], Wenqing Zhang[3], Hong Wang[2, 4, 5, *]

[1]State Key Laboratory for Mechanical Behavior of Materials, School of Material Science and Engineering, Xi'an Jiaotong University, Xi'an 710049, People's Republic of China

[2]School of Electronic and Information Engineering and State Key Laboratory for Mechanical Behavior of Materials, Xi'an Jiaotong University, Xi'an 710049, People's Republic of China

[3]Department of Physics, Southern University of Science and Technology, Shenzhen 518055, People's Republic of China

[4]Department of Materials Science and Engineering, Southern University of Science and Technology, Shenzhen 518055, People's Republic of China

[5]Shenzhen Engineering Research Center for Novel Electronic Information Materials and Devices, Southern University of Science and Technology, Shenzhen 518055, People's Republic of China

*Corresponding authors. E-mail: fanghj@xjtu.edu.cn (Huajing Fang); wangh6@sustech.edu.cn (Hong Wang)

S1 Synthesis of $Ti_3C_2T_x$ (MXene)

The MXene aqueous solution was fabricated via etching, intercalation and delamination. Etching: 5 g Ti_3AlC_2 powders (MAX phase) (≤38 μm particle size, Jilin 11 Technology Co., Ltd, China) were mixed in 60 mL of aqueous hydrofluoric acid (40%, Aladdin, China) for 24 h magnetic stirring at room temperature. And the produced residue was washed separately eight times with deionized water by centrifugation (10 min per cycle at 8000 rpm). Then the residue was dried in vacuum at 80 °C for 24 h. Intercalation: The dried HF-etched $Ti_3C_2T_x$ powders were mixed in dimethyl sulfoxide (DMSO) for intercalation. The DMSO/ $Ti_3C_2T_x$ solution ratio is 1 g of $Ti_3C_2T_x$ for 60 mL of DMSO. Then the solution was stirred with a magnetic stir bar for 24 h at room temperature, then centrifuged at 3500 rpm for one hour to separate DMSO from the sediment. Moreover, the sediment was washed with deionized water to remove the remaining DMSO. Delamination: Then the obtained sediment was added into 50 mL of deionized water and sonicated in ice-water bath for

1 h. Then the dispersion was centrifuged at 3500 rpm for 1 h to collect the dark supernatant. Finally, the stable MXene $Ti_3C_2T_x$ nanoflakes in aqueous solution were obtained.

S2 Supplementary Figures

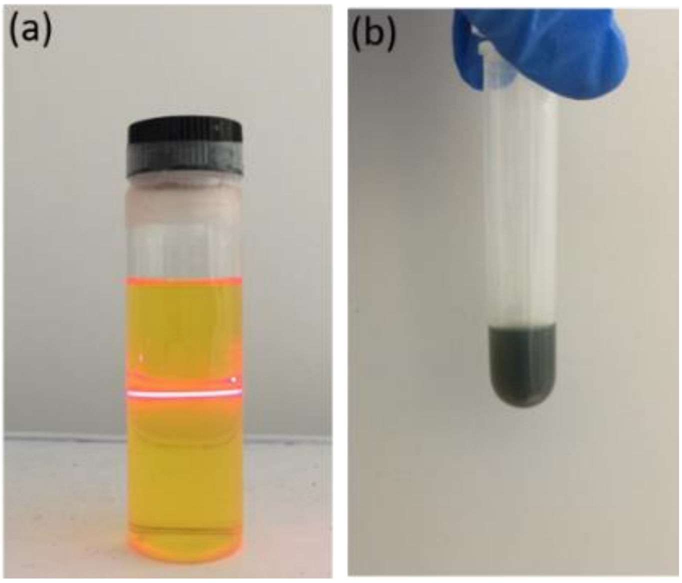

Fig. S1 a Clear yellow tungsten oxide precursor **b** Hybrid brownish green solution of MXene/tungsten oxide

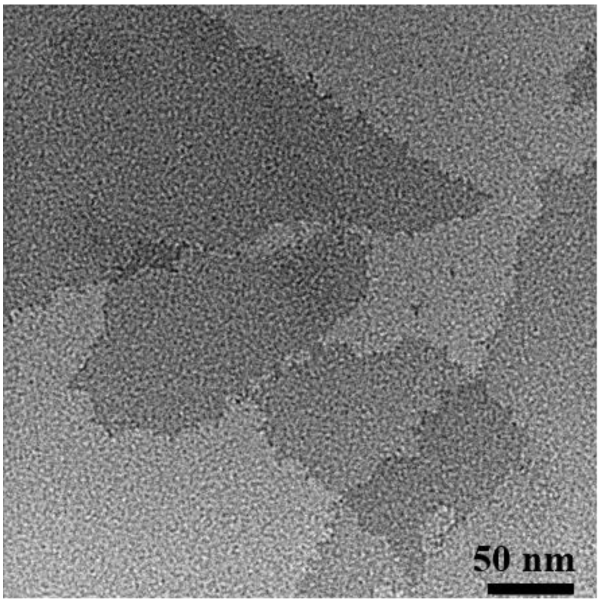

Fig. S2 TEM image of the MXene from top view confirming the separated two-dimensional nanoflakes

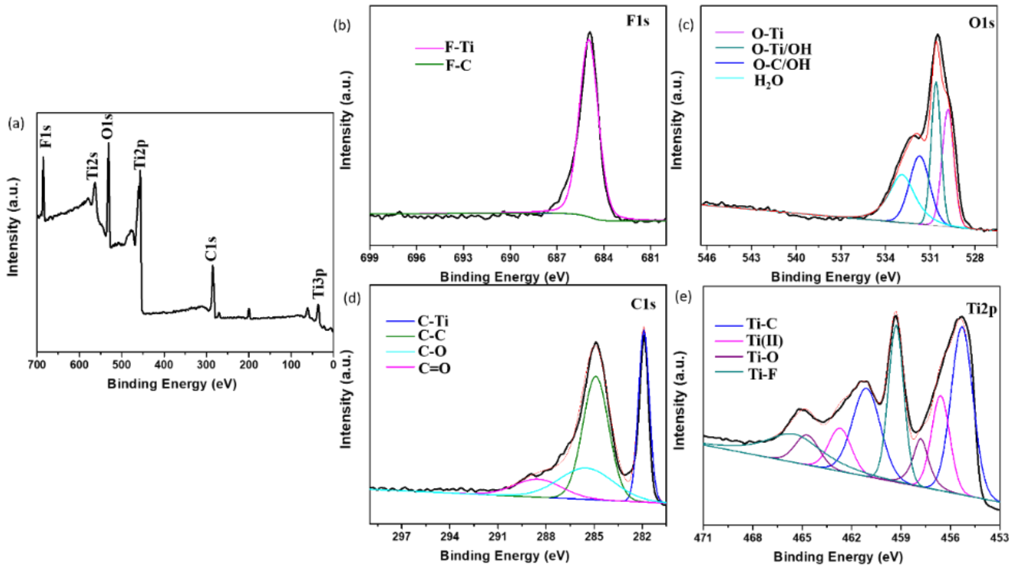

Fig. S3 XPS results of (**a**) XPS survey, (**b**) F 1s, (**c**) O 1s, (**d**) C 1s, and (**e**) Ti 2p of $Ti_3C_2T_x$ (MXene)

The XPS survey in Fig. S3a shows that F, O, C, and Ti elements are found in MXene, which is consistent with our fabrication process. Figure S3b shows two peaks (F-Ti and F-C) in the XPS spectra for F1s, confirming the presence of F functional group. In the O 1s spectrum (Fig. S3c), we have observed the formation of titanium oxide and verified the existence of -OH functional group. In the XPS spectra for C 1s (Fig. S3d), there are four peaks at 281.9, 284.9, 285.5, and 288.5 eV, corresponding to C-Ti, C-C, C-O, and C=O bonds [S1]. As seen from the Ti 2p spectrum (Fig. S3e), there are four peaks occurring at 455.3/461.1 eV, 456.6/464.7 eV, 457.8/464.7 eV, and 459.3/465.5 eV, which are corresponding to the Ti-C, Ti(II), Ti-O, and Ti-F bonds, respectively [S2]. In brief, the XPS results confirm the existence of H, F, O, C, Ti elements and the presence of -OH and -F functional groups in MXene.

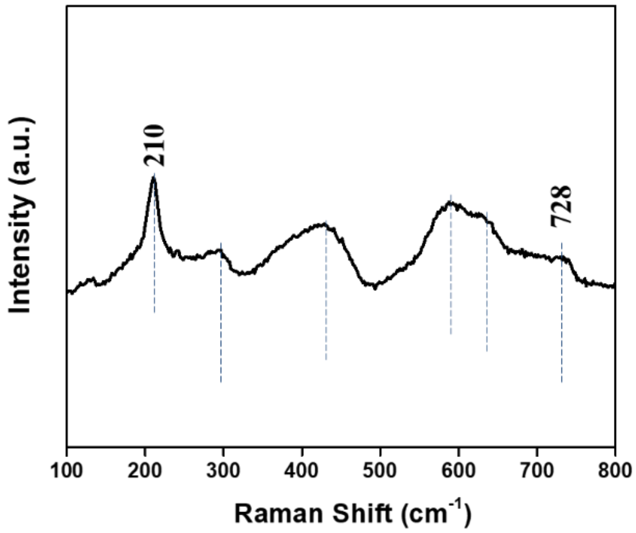

Fig. S4 Raman spectra of MXene

The Raman spectra characterizations of the MXene are shown in Fig. S4. Overall spectra trend keeps consistent with the reported works [S1, S2]. According to the Raman study of MXene [S3], the Raman peak at 210 cm^{-1} is assigned to the out-of-plane vibration mode (A_{1g}) of Ti, O, and C atoms, and the peak at 728 cm^{-1} is indexed to A_{1g}(C). The locations of these peaks are acceptable in the related study. The region 230-500 cm^{-1} reflects in-plane vibrations mode (E_g) of surface groups to Ti atom. And Raman bands between 580 and 730 cm^{-1} are assigned mostly to both E_g and A_{1g} modes to C atom.

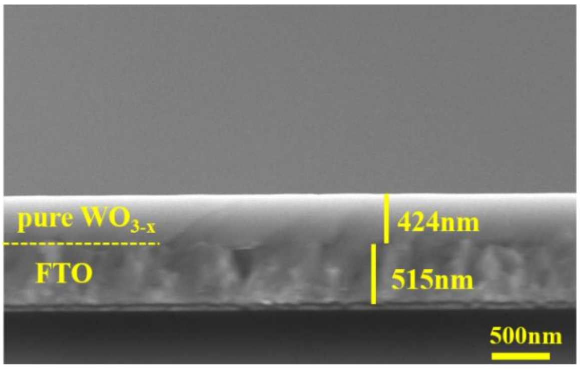

Fig. S5 Cross-sectional SEM image of pure WO_{3-x} film

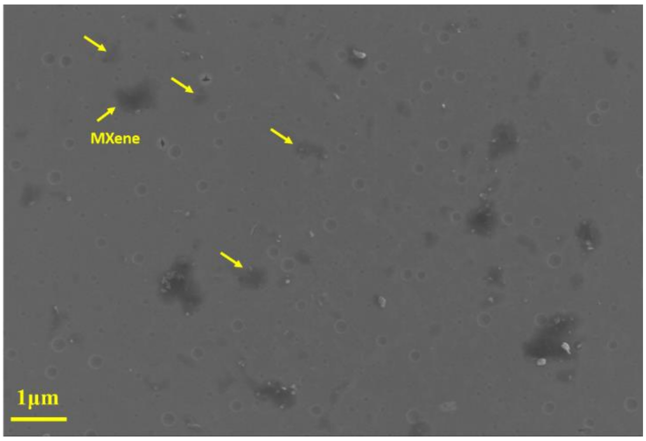

Fig. S6 Surface SEM image of the MXene/WO_{3-x} composite film

There are no crystal particles in the substrate, indicating the amorphous WO_{3-x} substrate. And small black MXene nanoflakes are found to distribute in the amorphous WO_{3-x} substrate, where the size varies from tens to hundreds of nanometers. The hemispherical concave holes are caused by the surface tension during the evolution processes of sol-gel systems [S4].

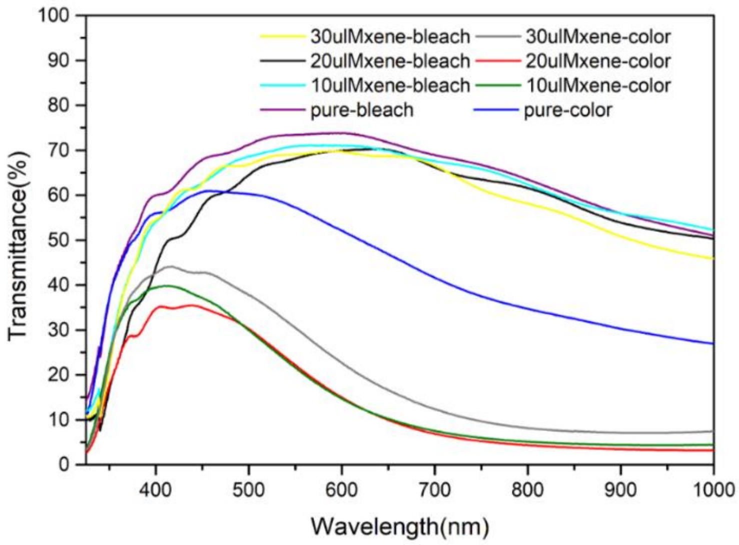

Fig. S7 Transmittance spectra of pure WO_{3-x} and 10/20/30 μL MXene/WO_{3-x} electrochromic devices in colored and bleached states

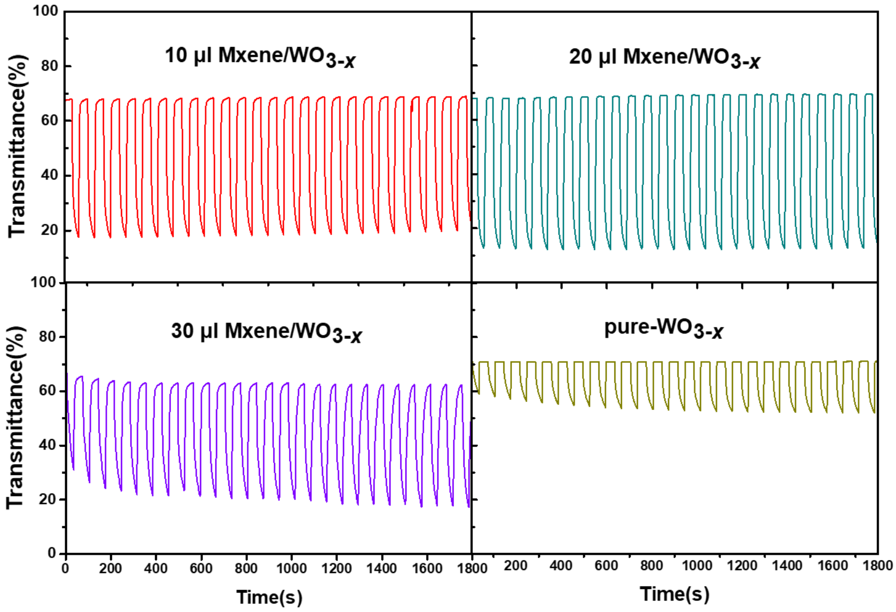

Fig. S8 Cyclic stability of the electrochromic devices based on pure WO_{3-x} film and 10/20/30 μL MXene/WO_{3-x} composite films at 660 nm

Different MXene additions of 10/20/30 μL are added into 2 mL of the tungsten oxide precursor to fabricated the 10 μL MXene/WO_{3-x}, 20 μL MXene/WO_{3-x} and 30 μL MXene/WO_{3-x} composite electrochromic films, respectively. The transmittance modulation and cycling performance are shown in Fig. S8 and Fig. S9. Compared with the pure WO_{3-x} film, composite films all exhibit enhanced electrochromic performances in varying degrees. And the 20 μL MXene/WO_{3-x} composite film exhibits largest transmittance modulation (60.4% at 660nm) and the most stable cycle performance. It's confirmed that 20 μL MXene: 2mLWO_{3-x} is the optimal ratio.

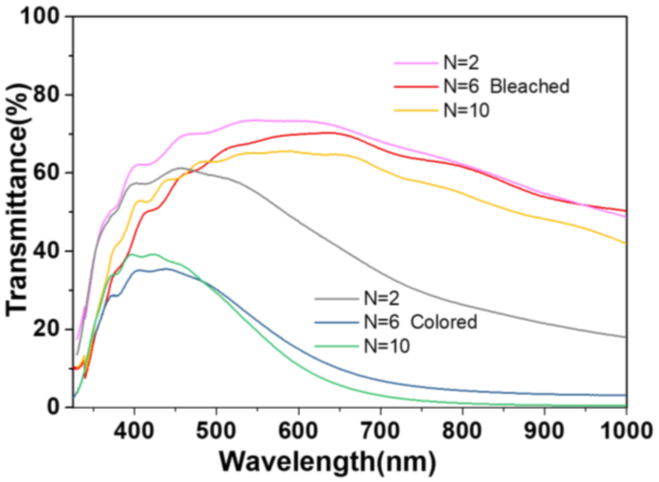

Fig. S9 Transmittance spectra of different thickness of N=2/6/10 of MXene/WO_{3-x} electrochromic devices in colored and bleached states

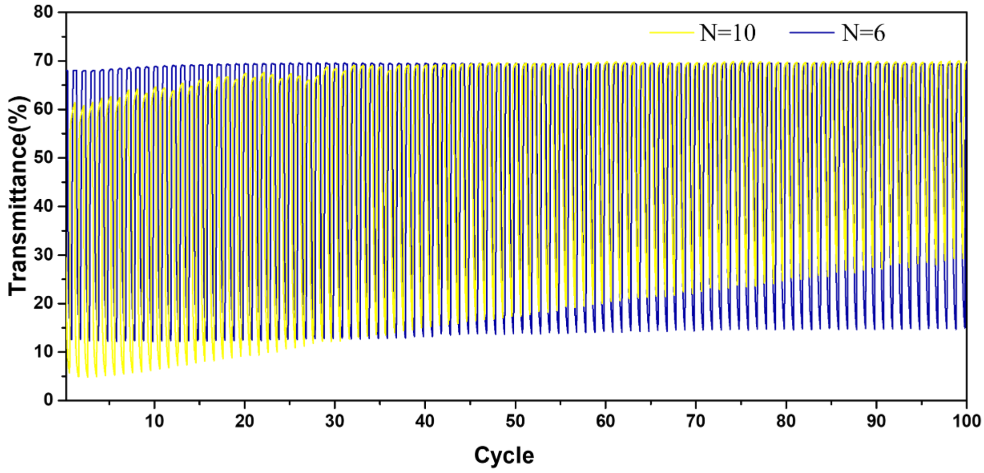

Fig. S10 Cyclic stability of the MXene/WO_{3-x} electrochromic devices with different thickness (N=6/10) at 660 nm

We regulate the thickness of the MXene/WO_{3-x} composite film by changing the layer number (N) at the same spin speed during spin-coating process. The thickness of 430 nm corresponds to N=6. And we have studied different thickness of N=2/6/10 on electrochromic performances of the MXene/WO_{3-x} composite film. As shown in transmittance spectra (Fig. S10), the MXene/WO_{3-x} composite film (N=2) shows small transmittance modulation, which is caused by a small amount of electrochromic material in the film. The decreased transmittance in bleached state in MXene/WO_{3-x} film (N=10) is caused by the increasing thickness of the film. And we further investigate the electrochromic cyclic stability (100 cycles) of MXene/WO_{3-x} film with different thickness (N=6/10), since the transmittance modulation of MXene/WO_{3-x} composite film (N=10) is comparable to that of N=6. It's clear that MXene/WO_{3-x} composite film of thickness of 430 nm (N=6) shows excellent stability (Fig. S11) and optimal overall electrochromic performances.

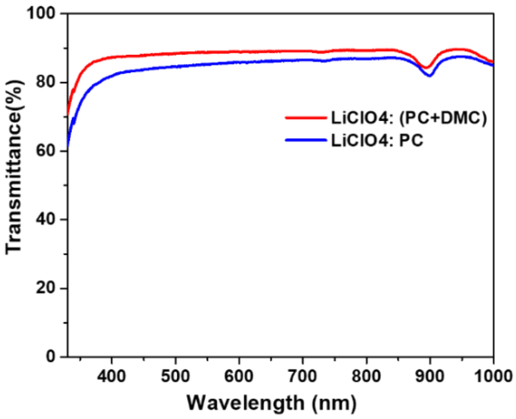

Fig. S11 Transmittance spectra of electrolytes

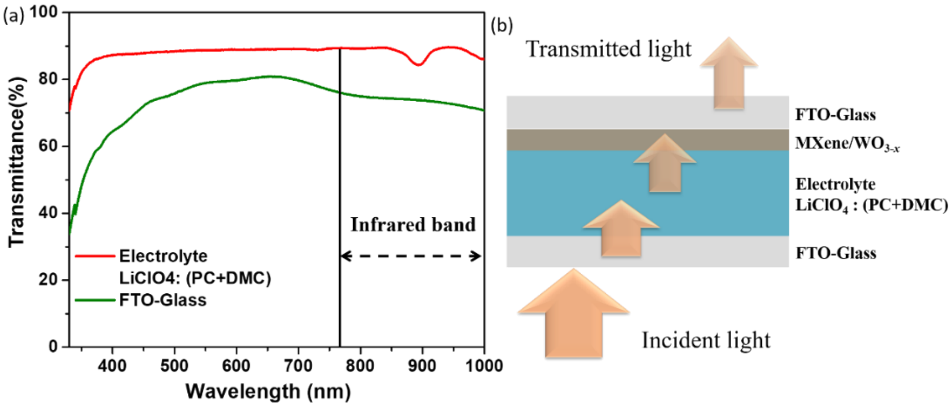

Fig. S12 a Transmittance spectra of electrolytes **b** Schematic explanation of the transmittance test in the bleached electrochromic device.

The transmittance of the device is influenced by the transmittance of FTO glass, electrolyte layer and electrochromic film. In the infrared band, the transmittance of FTO glass and electrolyte is around 70% and 90%, respectively. Therefore, the incident light is gradually weakened by the all layers in the device (Fig. S12b), showing a transmittance of lower than 60% in the infrared band in the bleached state.

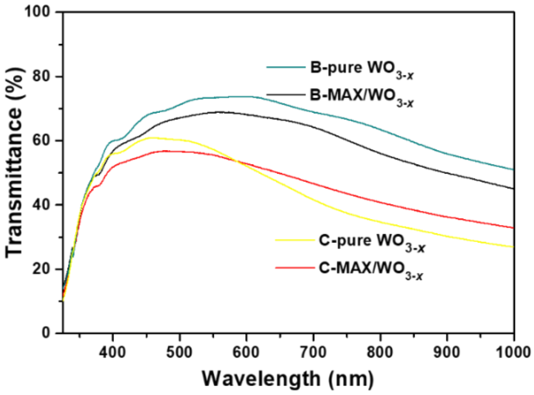

Fig. S13 Transmittance spectra of MAX/WO_{3-x} and pure WO_{3-x} device in colored and bleached states

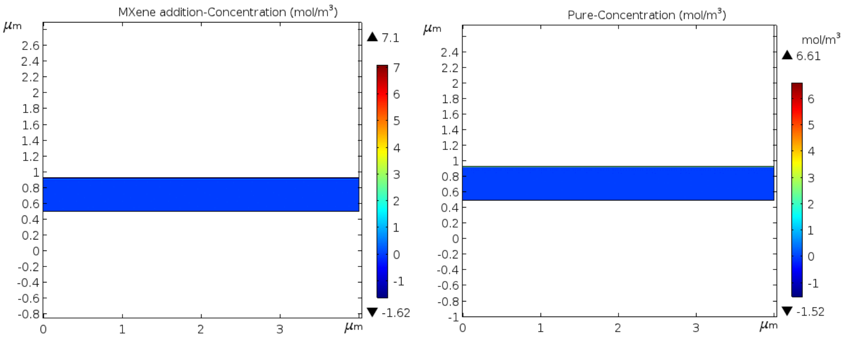

Fig. S14 Two pictures with gif format show the dynamic lithium ion diffusion process in the electrochromic layer (left-MXene/WO_{3-x}, right-pure WO_{3-x})

The pictures show spatial distribution of dynamic lithium ion concentration at each moment in the whole coloration time (30 s). Compared the MXene/WO_{3-x} film with the pure WO_{3-x} film, the MXene/ WO_{3-x} composite electrochromic film presents higher lithium concentration in the whole area. It further indicates the faster lithium ion diffusion speed in the composite film.

Supplementary References

[S1] G. Liu, J. Shen, Q. Liu, G. Liu, J. Xiong et al., Ultrathin two-dimensional MXene membrane for pervaporation desalination. Journal of Membrane Science **548**, 548-558 (2018). https://doi.org/10.1016/j.memsci.2017.11.065

[S2] J. Zhang, N. Kong, S. Uzun, A. Levitt, S. Seyedin et al., Scalable manufacturing of free-standing, strong $Ti_3C_2T_x$ MXene films with outstanding conductivity. Adv. Mater. **32**(23), 2001093 (2020). https://doi.org/10.1002/adma.202001093

[S3] A. Sarycheva, Y. Gogotsi, Raman spectroscopy analysis of the structure and surface chemistry of $Ti_3C_2T_x$ MXene. Chem. Mater. **32**(8), 3480-3488(2020). https://doi.org/10.1021/acs.chemmater.0c00359

[S4] R. Xu, E.J.A. Pope, J.D Mackenzi, Structural evolution of sol-gel systems through viscosity measurement. J. Non-Crystalline Solids **106**(1), 242-245 (1988). https://doi.org/10.1016/0022-3093(88)90267-0
